# The Effect of *BCMO1* Gene Variants on Macular Pigment Optical Density in Young Healthy Caucasians

**DOI:** 10.3389/fnut.2014.00022

**Published:** 2014-12-04

**Authors:** Zachary Kyle-Little, Andrew J. Zele, C. Phillip Morris, Beatrix Feigl

**Affiliations:** ^1^Medical Retina Laboratory, Institute of Health and Biomedical Innovation (IHBI), Queensland University of Technology, Brisbane, QLD, Australia; ^2^School of Biomedical Sciences, Queensland University of Technology, Brisbane, QLD, Australia; ^3^School of Optometry and Vision Science, Queensland University of Technology, Brisbane, QLD, Australia; ^4^Queensland Eye Institute, South Brisbane, QLD, Australia

**Keywords:** macular pigment optical density, BCMO1, macular carotenoids, lutein, zeaxanthin, macula

## Abstract

**Background:** Serum lutein (L) and zeaxanthin (Z) positively correlate with macular pigment optical density (MPOD); hence, the latter is a valuable indirect tool for measuring L and Z content in the macula. L and Z have been attributed antioxidant capacity and protection from certain retinal diseases but their uptake within the eye is thought to depend on genetic, age, and environmental factors. In particular, gene variants within beta-carotene monooxygenase (BCMO1) are thought to modulate MPOD in the macula.

**Objectives:** To determine the effect of *BCMO1* single nucleotide polymorphisms (SNPs) rs11645428, rs6420424, and rs6564851 on MPOD in a cohort of young healthy participants of Caucasian origin with normal ocular health.

**Design:** In this cohort study, MPOD was assessed in 46 healthy participants (22 male and 24 female) with a mean age of 23.8 ± 4.0 years (range 19–33). The three SNPs, rs11645428, rs6420424, rs6564851 that have established associations with MPOD were determined using MassEXTEND (hME) Sequenom assay. One-way analysis of variance was performed on groups segregated into homozygous and heterozygous *BCMO1* genotypes. Correlations between body mass index (BMI), iris color, gender, central retinal thickness (CRT), diet, and MPOD were investigated.

**Results:** Macular pigment optical density neither significantly varied with *BCMO1* rs11645428 (*F*_2,41_ = 0.70, *p* = 0.503), rs6420424 (*F*_2,41_ = 0.21, *p* = 0.801) nor rs6464851 homozygous or heterozygous genotypes (*F*_2,41_ = 0,13, *p* = 0.88), in this young healthy cohort. The combination of these three SNPs into triple genotypes based on plasma conversion efficiency did not affect MPOD (*F*_2,41_ = 0.07, *p* = 0.9). There was a significant negative correlation with MPOD and CRT (*r* = −0.39, *p* = 0.01) but no significant correlation between BMI, iris color, gender, and MPOD.

**Conclusion:** Our results indicate that macular pigment deposition within the central retina is not dependent on *BCMO1* gene variants in young healthy people. We propose that MPOD is saturated in younger persons and/or other gene variant combinations determine its deposition.

## Introduction

Macular pigment is composed of the xanthophyll carotenoids, lutein (*L*), zeaxanthin (*Z*), and meso-zeaxanthin (*MZ*) and has protective functions including those as an antioxidant and short-wavelength (blue) light filter within the central retina (1). Macular pigments cannot be synthesized *de novo* and must be acquired via dietary means, whereas *MZ* is produced as a metabolite of *L* within the retina (2). Dietary intake of xanthophylls rich foods, serum concentration of *L* and *Z*, and macular pigment optical density (MPOD) are all positively correlated in healthy samples (3). It is widely agreed that accumulation of macular pigment within the central retina depends on a range of biological processes including intestinal absorption, transport in serum, and retinal capture. A complete understanding of these processes, however, remains elusive (4). Using dietary supplements to influence the concentration of these carotenoids in the plasma and within the retina has been extensively studied, but with conflicting results as to the effect of dietary supplementation on MPOD and protection from age-related macular degeneration (AMD) (5–9).

Current literature supports the hypothesis that there is no single cause for the rate of macular pigment deposition but rather the interaction of dietary (10), genetic (11), and environmental factors control macular pigment deposition in an individual (3, 6). Environmental factors include diet, body mass index (BMI), and smoking status. For example, Mares et al. (12) reported that higher than normal abdominal body fat correlated strongly with lower MPOD levels. Additional research by Nolan et al. (13) identified that participants with a family history of AMD, heavy smokers, and those with a BMI >27 did not show a relationship with serum concentrations of L and Z and MPOD whereas participants without a risk for AMD showed a positive correlation between serum concentration and MPOD. The researchers proposed that individuals at risk for AMD may have compromised function in retinal capture of macular pigments or macular deposition. Heritable factors such as iris color and ethnicity may also be important, with a positive correlation between darker iris colors and higher MPOD (14).

The critical role of gene variants in the determination of MPOD has only recently received attention among the scientific community (15–18), with further research being essential to develop a complete understanding of the pathway to macular pigment deposition. The main enzyme for vitamin A metabolism is the enzyme beta-carotene monooxygenase (BCMO1), which cleaves the non-macular carotenoid beta-carotene (pro-vitamin A) to produce two identical molecules of retinal (vitamin A) (19). As such, the cleavage efficiency of BCMO1 is thought to mediate the competition of macular and non-macular carotenoids for absorption (20); a high BCMO1 conversion efficiency results in a lower plasma beta-carotene, hence, higher macular carotenoid concentration available for deposition within the central retina. On the other hand, low-BCMO1 conversion efficiency results in a higher plasma beta-carotene hence less macular carotenoid available for retinal deposition. Various single nucleotide polymorphisms (SNPs) within the *BCMO1* gene have been implicated to modulated BCMO1 action (21, 22) with homozygous genotypes of either of the three *BCMO1* SNPs rs11645428, rs6420424, and rs6564581 having reduced or increased BCMO1 catalytic activity, resulting in an increase or decrease in beta-carotene plasma concentration (22). Homozygous rs6564581 G allele carriers have higher beta-carotene but lower lutein and zeaxanthin plasma levels (21). There is an established association between *BCMO1* SNPs and MPOD (6, 15, 17) but it is not well understood how genotypes with high- or low-BCMO1 conversion efficiency affect MPOD, in particular, in persons of different age, gender, and ethnicity. Feigl et al. (16) provided the initial demonstration of the effect of the *BCMO1* SNPs rs11645428, rs6564851, and rs6420424 on MPOD variations in a mixed gender group aged over 50 years. They demonstrated that participants with rs11645428 GG, rs6564851 GG, and rs6420424 AA genotypes had lower MPOD compared to the other homozygous and heterozygous *BCMO1* genotypes (16). However, this was not evident in patients with manifest AMD and may also vary with age (23). The objective of this study was therefore to investigate *BCMO1* rs11645428, rs6564851, and rs6420424 genotypes in young healthy participants (≤35 years) to determine whether the single SNP genotypes or their combinations can explain the variation in MPOD in a younger cohort.

## Materials and Methods

### Participants

Participants were recruited in the study according QUT’s human ethics approval. Participants were included if they were aged between 19 and 33 years, female or male, of normal general and ocular health and excluded if they had a color vision deficiency or were taking supplements containing L or Z. Of the 48 participants who volunteered, one was excluded due to protanopic congenital color vision deficiency and one due to zeaxanthin supplementation. This resulted in a sample of 46 participants eligible for further testing. All recruitment and experiments were conducted in accordance with the QUT Human Research Ethics Committee (QUT ethics approval number 1300000089) and the tenets of the declaration of Helsinki.

To determine the general health and well-being of the study cohort the familial and personal medical history was taken and the BMI was calculated (24). Because dietary intake of L and Z is positively correlated with MPOD a 7-day food recall survey, adapted from a validated food frequency questionnaire (24), was undertaken to determine the consumption frequency of fast foods, fruit, vegetables, and eggs each week. Scores of 1, 2, or 3 were assigned according to the published procedures (24). An ophthalmic examination was performed in all patients including visual acuity, intraocular pressure (i-care, Finland), and optical coherence tomography (OCT) (FD-OCT, Cirrus, Zeiss Oberkochen, Germany) in accordance with standard procedures. All participants had normal ocular health.

A macular pigment densitometer (Macular Metrics II, LLC, Providence, RI, USA) was used to perform heterochromatic flicker photometry (HFP). The protocol followed the standardized protocol for measuring MPOD by HFP (25) and as previously used in our laboratory (16, 26). All participants were considered naive to the HFP procedure and underwent brief training and practice trials before completing the task.

To determine the SNPs within the *BCMO1* gene of each participant, a 2 mL saliva sample was collected with Oragene™ self-collection kits (OG-500, Genotek, Canada). Samples were stored at −80°C before manual DNA purification, which was performed according to the manufacturer’s protocol and Australian Genome Research Facility (AGRF) (Brisbane, QLD, Australia) guidelines. *BCMO1* SNPs rs11645428 (SNP1), rs6420424 (SNP2), and rs6564851 (SNP3) were genotyped using a commercial genotyping service provided by the AGRF. The method used employed matrix-assisted laser desorption/ionization time-of-flight mass spectrometry to genotype using a homogenous MassEXTEND™ assay (Sequenom, San Diego, CA, USA).

### Data analysis

All statistical analysis was performed using the software SPSS version 20 (SPSS Inc., Chicago, IL, USA). Initial screening indicated all data met the assumptions of the statistical tests. To determine whether there was a significant difference between the three SNP genotypes, either singly or in combination, and MPOD, a one-way analysis of variance (ANOVA) and *post hoc* analysis where necessary, were performed. In addition, a Pearson correlation was performed to determine the relationship between MPOD, gender, ethnicity, central retinal thickness (CRT), BMI, and iris color. A *p*-value of 0.05 was considered as statistically significant.

## Results

Genotype frequencies were determined for rs11645428, rs6420424, and rs6564851 (Table 1). Four samples failed the genotyping assay, resulting in 42 samples for further analysis. Out of the 42 participants, 19 were male and 23 were female (mean age = 23.8 years ± 4.0 SD). Thirty-eight participants (91%) were in a healthy weight range, none were underweight or obese, and four (9%) were overweight. The average BMI of the cohort was 22.40 ± 2.4 kg. Ethnicity was identified by self-report; 33 (79%) participants identified European descent and 9 (21%) identified Asian descent. Smoking status was self reported; five participants, or 14% of the cohort, identified as casual or habitual smokers.

**Table 1:** ***BCMO1* rs11645428, rs6420424, and rs6564851 genotype frequencies**.

rs11645428 (SNP1)		rs6420424 (SNP2)		rs6564851(SNP3)	
GG	16 (38%)	AA	10 (24%)	GG	8 (19%)
AG	19 (45%)	GA	20 (48%)	GT	21 (50%)
AA	7 (17%)	GG	12 (28%)	TT	13 (31%)

**Figure 1:**
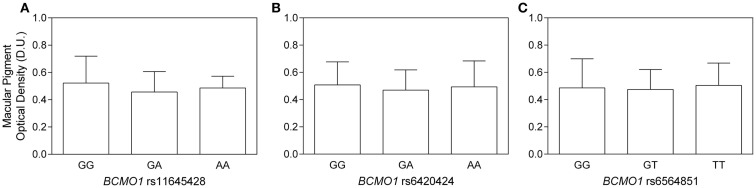
**(A–C)** Macular pigment optical density as a function of **(A)** *BCMO1* rs11645458, **(B)** *BCMO1* rs6450424, and **(C)** *BCMO1* rs6564851 genotypes (error bars indicate ±SD). There is no significant difference in MPOD between homozygous and heterozygous genotypes.

For *BCMO1* SNP rs11645428, the mean MPOD for the homozygous G allele was 0.52 ± 0.2 D.U.; the homozygous A allele was 0.49 ± 0.1 D.U.; and the heterozygous AG allele was 0.46 ± 02 D.U. (Figure 1A). There was no significant difference in MPOD between rs11645428 AA, AG, and GG genotypes (*F*_2,41_ = 0.700, *p* = 0.503). For the *BCMO1* SNP rs6420424, the mean MPOD for the homozygous A allele was 0.50 ± 0.2 D.U., the homozygous G allele was 0.51 ± 0.2 D.U., and the heterozygous GA allele, 0.47 ± 0.2 D.U. (Figure 1B). There was no significant difference in MPOD between rs6420424 AA, GA, and GG genotypes (*F*_2,41_ = 0.210, *p* = 0.81). The mean MPOD for the homozygous T, G, and heterozygous GT allele for the *BCMO1* SNP rs6564851 were 0.50 ± 0.2 D.U., 0.49 ± 0.2 D.U., and 0.47 ± 0.2 D.U., respectively (Figure 1C) and there was no significant difference between alleles (*F*_2,41_ = 0,13, *p* = 0.88).

**Figure 2:**
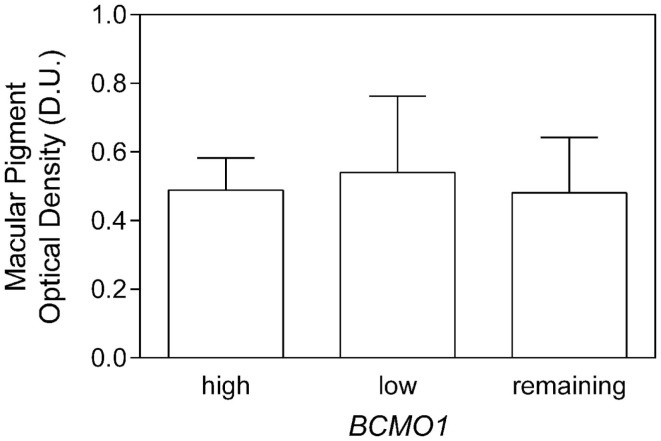
**Macular pigment optical density as a function of high- and low-BCMO1 plasma conversion triple genotypes and remaining genotypes**. There was no significant difference between MPOD within the genotypes.

While there was no significant effect of single *BCMO1* SNPs on MPOD, we investigated triple genotype combinations based on *BCMO1* plasma conversion efficiency as it has been investigated in a mixed cohort of participants over the age of 50 years (16). The triple genotypes were combined as follows; high conversion (SNP1 AA/SNP2 GG/SNP3 TT), low conversion (SNP1 GG/SNP2 AA/SNP3 GG), and medium conversion (remaining SNP1, SNP2, and SNP3 genotypes) frequencies are given in Table 2. The mean MPOD for high, low, and medium triple BCMO1 conversion genotypes were 0.49 ± 0.2 D.U., 0.54 ± 0.2 D.U., and 0.48 ± 0.1 D.U., respectively (Figure 2), which were not significantly different (*F*_2,41_ = 0.07, *p* = 0.9).

**Table 2:** ***BCMO1* triple genotype frequencies and MPOD**.

Triple genotypes	SNP1AA/SNP2GG/SNP3TT	SNP1GG/SNP2AA/SNP3GG	Remaining genotypes
Frequency	6 (14%)	7 (16%)	29 (69%)
MPOD D.U. ± SD	0.49 ± 0.2	0.54 ± 0.2	0.48 ± 0.1

A Pearson correlation showed that MPOD was not significant correlated with gender (*p* = 0.3), BMI (*p* = 0.7), iris color (*p* = 0.8), and smoking status (*p* = 0.5) whereas CRT was negatively correlated (*r* = −0.39, *p* = 0.01). The results of 36 participants who completed the dietary questionnaire demonstrated that participants with the lowest dietary score (score 1; *n* = 3) had on average a lower MPOD (0.30 D.U. ± 0.21) compared to those participants with the highest score (score 5; *n* = 5) who had an MPOD of 0.55 D.U. ± 0.13. The remaining participants with scores 2 (*n* = 8), 3 (*n* = 15), and 4 (*n* = 5) had an average MPOD of 0.5 D.U. ± 0.15, 0.46 D.U. ± 0.15, and 0.48 D.U. ± 0.1, respectively.

## Discussion

The results show that in a mixed cohort of younger participants (≤33 years of age), there was no significant difference in MPOD between the three *BCMO1* SNPs rs11645428, rs6420424, and rs6564851 that have been shown to affect MPOD in older persons (16). Our results further demonstrate that MPOD does not significantly vary between “high-plasma conversion,” “medium,” and “low conversion” triple *BCMO1* genotypes. This suggests that these *BCMO1* SNPs do not play a significant role in determining MPOD in this young, healthy cohort.

Feigl et al. (16) demonstrated a significant effect of each of these three SNP genotypes and their combinations on MPOD levels in an older cohort (Mean age: 56 ± 5 years) in support of the hypothesis that *BCMO1* mediates macular pigment uptake. The observation that *BCMO1* SNP genotypes correlated with MPOD variation in older healthy participants but not in the younger cohort in the current study, indicates that age may have an effect on macular pigment transport and/or deposition mechanisms.

**Figure 3:**
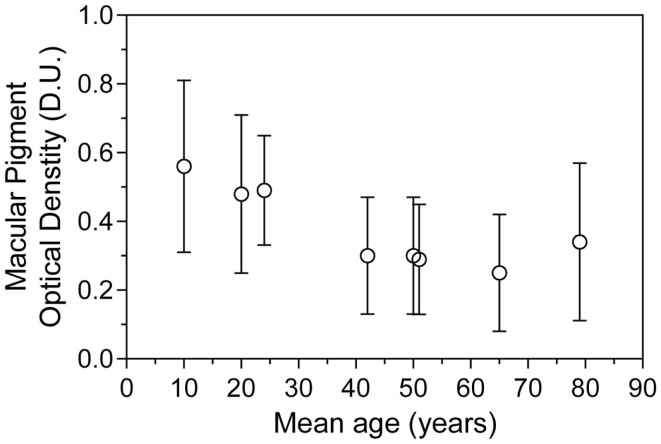
**Mean macular pigment optical density as a function of mean age ± SD, collated from eight representative studies of heterochromatic flicker photometry**.

The point of MPOD saturation or maximal MPOD in a given cohort has been observed in numerous supplementation studies (5, 27) but remains variable between individuals (28). Moreover, differences between study protocols, especially participant age, macular pigment supplement formulation, and the length of study, may make finding an appropriate mean value for saturated MPOD difficult to predict. Over the last decade, numerous studies of MPOD have been carried out in many age groups (Table 3). Figure 3 plots the MPOD values from these studies listed in Table 3 as a function of the cohort age and the mean MPOD decreases with age. Our findings support the hypothesis that in younger cohorts, the macular pigment may be saturated in the macula and therefore any competition for absorption would be inconsequential. On the other hand, older cohorts with lower mean MPOD would be affected by the rate of competition for macular pigment deposition within the central retina. We postulate that in younger age groups, BCMO1 may not be the rate determining factor of macular pigment density. This macular pigment saturation hypothesis is supported by the results from a study by Yonova-Doing et al. (6). In their sample of 310 healthy twins, the *BCMO1* SNP rs11645428 was correlated with baseline MPOD prior to supplementation. However, after supplementation of *L* and *Z*, this polymorphism was no longer significantly affecting MPOD. This may indicate that the threshold for a *BCMO1* effect on MPOD had been surpassed with the increased levels of *L* and Z, similar to a young cohort with a healthy diet as apparent in our current study.

**Table 3:** **Studies measuring MPOD (HFP, 1° foveal stimulus), ordered according to increasing age**.

Study	Sample size	Age range and/or mean ± SD	Mean MPOD ± SD
Zheng et al. (29)^a^	94	6–12 years, 9.5 ± 1.63 years	0.56 ± 0.25
Tang et al. (30)^b^	67	18–23 years	0.48 ± 0.23
***Current Study***^a^	***42***	***19–33, 23.8* ***±* ***4 years***	***0.49* ***±* ***0.16***
Nolan et al. (31)^a^	800	20–60, 41.94 ± 11.62	0.30 ± 0.17
Berendschot and van Norren (32)^b^	53	50 ± 16 years	0.30 ± 0.17
Beatty et al. (33)^b^	46	51 ± 18 years	0.29 ± 0.16
Nolan et al. (34)^b^	79	18–60, 65 ± 11 years	0.25 ± 0.17
Iannaccone et al. (35)^a^	222	79.1 ± 3.2 years	0.34 ± 0.23

*^a^Parafoveal stimulus 7°*.

*^b^Parafoveal stimulus between 4° and 6°*.

The carotenoids *L* and *Z* accumulate in the macula, serving as a “sink” (36) to the exception of all other non-macular carotenoids and their highly selective uptake is indicative of one or more specific transport and/or binding proteins. It is possible that a combination of these transporter proteins and receptors may be responsible for the variation in MPOD seen between younger and older cohorts. Recent findings on intracellular macular carotenoid transport mechanisms and selective retinal binding proteins (36–38) have produced insightful results, suggesting numerous locations where MPOD variance may be introduced between individuals that may be largely genetically determined (4, 11, 18, 39). Macular carotenoids are believed to share an absorption pathway with cholesterol and the cholesterol transporter protein, scavenger receptor class B, member 1 (SCARB1), plays a role in their intracellular uptake in the intestines (40). In particular, SNPs within the *SCARB1* gene modulate lutein uptake (40). We also investigated the three *SCARB1* SNPs; rs5888, rs10744182, and rs838879 in a pilot study (unpublished data) that were previously associated with carotenoid metabolism and MPOD (6, 17, 41). In this pilot, there was no significant difference in MPOD between *SCARB1* SNP rs5888 and rs10744182 genotypes but in a small sample size (*n* = 3) the SNP rs838879 affected MPOD in such way that participants with the homozygous G allele had on average higher MPOD compared to the homozygous A and heterozygous GA allele. The number of participants with the GG genotype, however, was low and further studies are needed to confirm whether *SCARB1* rs838879 GG is associated with higher MPOD.

We found a significant negative correlation between CRT and MPOD as previously reported (42) with higher MPOD values in persons with thinner CRT. Whether this can be related to a ring-like structure of MPOD distribution cannot be determined in this study as MPOD was only measured centrally with a 1° stimulus. Gender, iris color, and smoking were not found to be associated with MPOD in the present study. Hammond et al. (43) found a significant relationship between current smoker frequency and MPOD in participants aged between 17 and 92 years, suggesting that heavier smoking (>10 cigarettes a day) was related to lower MPOD. Given this younger cohort had only six smokers, we do not have sufficient statistical power to draw a conclusion. Overall participants with the lowest dietary score had on average lower MPOD (0.30 D.U. ± 0.21) compared to those with higher dietary scores. However, this survey is limited as there was a low number of participants with low scores and a larger number of participants with a healthy balanced diet, supporting the saturation hypothesis.

In summary, this is the initial demonstration that in a mixed gender cohort of young participants aged ≤33 years, *BCMO1* SNP genotypes do not explain variations in MPOD, whereas they have previously been shown to play a role in MPOD in older participants (16). We propose that the rate determining factor of macular pigment deposition within the retina is dependent on different genetic factors between young and older cohorts. The determining factors of MPOD in older participants may be related to carotenoid uptake whereas for young participants, MPOD may be determined by transport proteins. Further investigation into the biochemical pathway of macular pigment deposition and its genetic determinants are essential to the goal of protecting the macula from oxidative stress and preventing the onset of AMD through macular pigment augmentation.

## Conflict of Interest Statement

The authors declare that the research was conducted in the absence of any commercial or financial relationships that could be construed as a potential conflict of interest.
